# Extreme Drug Tolerance of Mycobacterium tuberculosis in Caseum

**DOI:** 10.1128/AAC.02266-17

**Published:** 2018-01-25

**Authors:** Jansy P. Sarathy, Laura E. Via, Danielle Weiner, Landry Blanc, Helena Boshoff, Eliseo A. Eugenin, Clifton E. Barry, Véronique A. Dartois

**Affiliations:** a Public Health Research Institute, New Jersey Medical School, Rutgers, The State University of New Jersey, Newark, New Jersey, USA; b Tuberculosis Research Section, Laboratory of Clinical Infectious Diseases, NIAID, NIH, Bethesda, Maryland, USA; c Department of Microbiology, New Jersey Medical School, Rutgers, The State University of New Jersey, Newark, New Jersey, USA; d Institute of Infectious Disease and Molecular Medicine, Department of Clinical Laboratory Sciences, University of Cape Town, Cape Town, South Africa; e Department of Medicine, New Jersey Medical School, Rutgers, The State University of New Jersey, Newark, New Jersey, USA

**Keywords:** Mycobacterium tuberculosis, persistence, drug tolerance, caseum, pharmacokinetics-pharmacodynamics, *in vitro* potency model

## Abstract

Tuberculosis (TB) recently became the leading infectious cause of death in adults, while attempts to shorten therapy have largely failed. Dormancy, persistence, and drug tolerance are among the factors driving the long therapy duration. Assays to measure *in situ* drug susceptibility of Mycobacterium tuberculosis bacteria in pulmonary lesions are needed if we are to discover new fast-acting regimens and address the global TB threat. Here we take a first step toward this goal and describe an *ex vivo* assay developed to measure the cidal activity of anti-TB drugs against M. tuberculosis bacilli present in cavity caseum obtained from rabbits with active TB. We show that caseum M. tuberculosis bacilli are largely nonreplicating, maintain viability over the course of the assay, and exhibit extreme tolerance to many first- and second-line TB drugs. Among the drugs tested, only the rifamycins fully sterilized caseum. A similar trend of phenotypic drug resistance was observed in the hypoxia- and starvation-induced nonreplicating models, but with notable qualitative and quantitative differences: (i) caseum M. tuberculosis exhibits higher drug tolerance than nonreplicating M. tuberculosis in the Wayne and Loebel models, and (ii) pyrazinamide is cidal in caseum but has no detectable activity in these classic nonreplicating assays. Thus, *ex vivo* caseum constitutes a unique tool to evaluate drug potency against slowly replicating or nonreplicating bacilli in their native caseous environment. Intracaseum cidal concentrations can now be related to the concentrations achieved in the necrotic foci of granulomas and cavities to establish correlations between clinical outcome and lesion-centered pharmacokinetics-pharmacodynamics (PK-PD) parameters.

## INTRODUCTION

The eradication of tuberculosis (TB) is hampered by long treatment duration due to the persistence of bacterial populations in selected niches. Effective treatment of drug-sensitive disease requires a combination of up to four drugs taken for 6 months. Despite such intensive therapeutic strategy, drug resistance is increasing worldwide and the disease relapses in 2% to 4% of patients under clinical trial conditions, with higher relapse rates in less-ideal settings (1). This relative inefficiency of anti-TB therapy is at odds with the pharmacokinetics-pharmacodynamics (PK-PD) of the two critical drugs in the regimen: rifampin and isoniazid. Concentrations of these two agents in plasma relative to their MIC, corrected for plasma protein binding, achieve targets that would be considered sufficient against other bacterial infections, most of which are cured within a week with a single antibiotic.

Why then is this not good enough for TB treatment? Two (non-mutually exclusive) theories have been considered to explain the need for the long and intensive therapy required to achieve cure (2). First, some bacterial populations are sequestered in remote lesion compartments which antibiotics fail to reach at adequate concentrations (3). Second, there exist recalcitrant subpopulations of bacteria that have become phenotypically drug tolerant in response to a variety of stresses ranging from drug exposure to immune pressure, nutrient shift, acidic pH, and low-oxygen tension (4, 5). A growing number of observations suggest that these two phenomena join forces to limit the efficacy of TB drugs. We have shown that drug distribution to the site of infection is both drug specific and lesion specific (6, 7); however, direct evidence demonstrating drug tolerance of Mycobacterium tuberculosis bacilli present inside lesions is lacking.

While the spectra of TB lesions are diverse, the persistence of TB disease appears to be driven by only a few key lesion types. The presence and extent of cavitary disease are associated with clinical treatment failure and relapse (8–10). In preclinical efficacy studies that focused on bacterial populations surviving drug treatment, the lesion compartments that failed to be sterilized at the end of therapy were mostly necrotic granulomas and caseous foci (11–14). Accordingly, we have recently shown that not all TB drugs diffuse effectively through the necrotic centers of cavities and of closed nodules, in which blood supply is absent. For example, moxifloxacin exhibits heterogeneous partitioning across the granuloma, with moderate to poor diffusion into the necrotic centers where oxygen tension is low compared to the surrounding macrophage layers (7, 15). Pyrazinamide (PZA) and rifampin, the two “sterilizing” drugs that have contributed most to treatment shortening (16), distribute favorably into all lesion compartments, including avascular caseum (7). Thus, favorable drug penetration into lesions, with an emphasis on caseous foci, seems to contribute to effective treatment.

However, this is only the pharmacokinetic aspect of lesion-centered pharmacology. To determine whether the concentrations of TB drugs in caseous cores are “adequate”—i.e., sufficient to kill the resident bacterial populations, including persistent bacilli with reduced drug susceptibility—one needs to know the potency of each drug against bacilli present in caseum. Several *in vitro* assays have been developed and used for that purpose (17), such as the Loebel starvation-induced model of nonreplication (18, 19) and the Wayne model of hypoxia-induced nonreplication (20). In an attempt to establish the predictive value of these assays developed to mimic persistence, we have measured the potency of first- and second-line agents against nonreplicating M. tuberculosis bacilli in *ex vivo* caseum. We have taken advantage of the rabbit model of active TB disease which exhibits many aspects of the human pathology, including the formation of cavities (21). Our results provide the first experimental evidence that M. tuberculosis bacteria present in caseum are largely slowly replicating or nonreplicating and display extreme drug tolerance, in support of the persistent-bacillus theory. This new factor may conspire with the factors of interindividual PK variability and suboptimal drug distribution into caseous lesions to create pockets of inadequate drug coverage, leading to relapse or emergence of drug resistance (3, 22). Together with our recent measurements of drug concentrations in various lesion types (7), the present results and associated assay will enable the calculation of true lesion-centered PK-PD parameters.

## RESULTS

### M. tuberculosis burden and growth in caseum.

**FIG 1:**
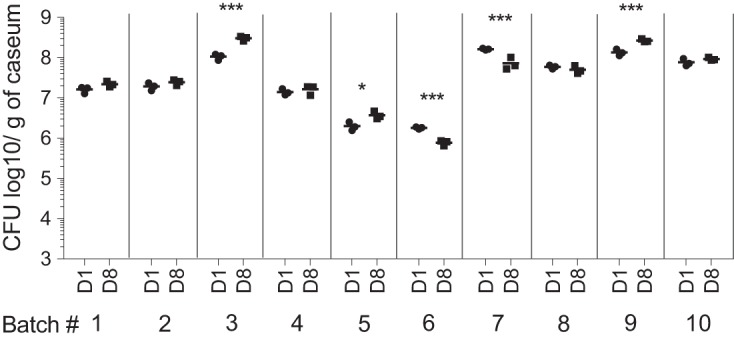
Bacterial burden of 10 different *ex vivo* samples of rabbit caseum incubated at 37°C for 7 days, measured on day 1 (D1) and day 8 (D8) in the absence of drug. Data are expressed as log_10_ of CFU per gram of caseum (undiluted) and plotted on a log scale. Statistically significant differences in CFU counts are indicated as follows: *, *P* < 0.05; **, *P* < 0.01; ***, *P* < 0.005.

We first asked whether M. tuberculosis bacilli residing within the caseum of rabbit cavities would replicate when incubated *ex vivo* at 37°C for 7 days, the intended duration of the cidal assay. Ten caseum samples were incubated independently, and CFU were enumerated in triplicate on day 1 and day 8. The samples were incubated under conditions of normal O_2_ tension in 96-well plates without shaking, likely resulting in a shallow gradient of O_2_ from the surface downward. This setup was used to mimic the conditions present at the surface of cavities. The bacterial burden recovered on day 1 ranged from 1 × 10^6^ to 2 × 10^8^ M. tuberculosis bacilli per gram of caseum. Using pH indicator strips, we determined that the pH of caseum homogenate (diluted 3-fold in water) on day 1 and day 8 ranged between 7.0 and 7.5. Since caseum samples were harvested from different lesions and often from different infected rabbits, the M. tuberculosis burden in each sample (thus the starting inoculum of each assay) was variable (Fig. 1). There was no significant change in bacterial burden over the 7-day incubation period for 6 of the 10 samples tested (*P* > 0.05). We observed a slight decrease for 2 samples and a slight increase for another 2 samples (Fig. 1); however, the overall difference in CFU levels between day 1 and day 8 was not significant (multiple *t* test). We also compared the net growth levels of M. tuberculosis in freshly explanted caseum and caseum stored at 4°C and incubated at 37°C and obtained similar results (see Fig. S1A in the supplemental material), indicating that the bacteria in the *ex vivo* caseum remained viable throughout the 7-day incubation period and were largely nongrowing. Thus, *ex vivo* caseum is an adequate tool to evaluate drug potency against M. tuberculosis bacilli in their native caseous environment.

### Reduced drug susceptibility of M. tuberculosis bacilli in caseum.

**FIG 2:**
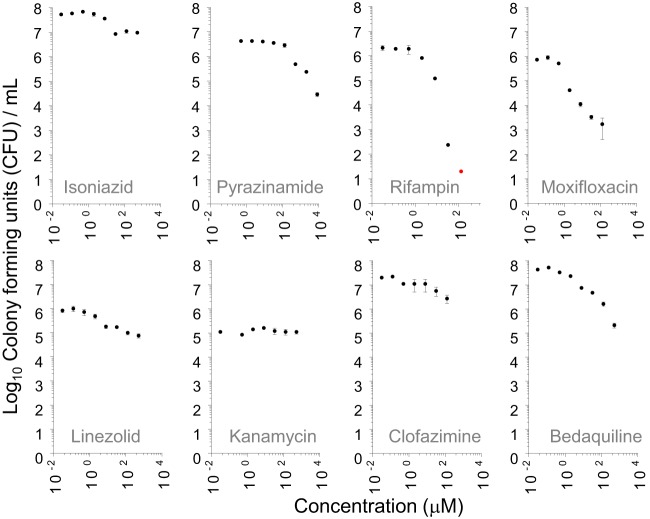
Bactericidal activity of eight standard TB treatment drugs in caseum. Data are expressed as log_10_ of average CFU per milliliter of homogenized caseum from three replicates. A red dot highlights data points below the limit of detection (LOD [approximately 20 CFU]); i.e., no CFU was recovered from the lowest dilution of caseum homogenate plated. Standard deviations are indicated by error bars.

**FIG 3:**
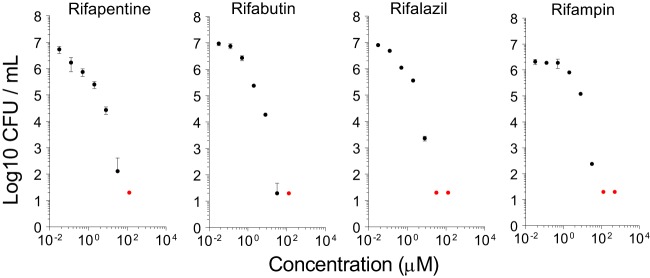
Bactericidal activity of four rifamycins in caseum. Data are expressed as log_10_ of average CFU per milliliter of homogenized caseum obtained from three replicates. Red dots highlight data points below the limit of detection (LOD [approximately 20 CFU]); i.e., no CFU was recovered from the lowest dilution of caseum homogenate plated. Standard deviations are indicated by error bars.

Next, we tested the cidal activity of seven first- and second-line anti-TB drugs under the same conditions, in dose-response format. Overall, intracaseum M. tuberculosis was highly tolerant of most antibiotics. Rifampin and moxifloxacin were most effective, with casMBC_90_ (concentrations in caseum required to kill 90% of the bacteria) of 8 μM and 2 μM, respectively (Fig. 2 and Table 1). Apparent sterilization was achieved by rifampin only, at 128 μM. Bedaquiline, linezolid, and pyrazinamide had modest activity against intracaseum M. tuberculosis, with casMBC_90_ values of 32, 128, and 512 μM, respectively. Isoniazid, kanamycin, and clofazimine had minimal to no activity; 90% killing was not achieved at any concentration tested. Isoniazid and rifampin were tested in caseum stored at 4°C and displayed the same casMBC_90_ value as that measured in frozen/defrosted caseum (not reached for isoniazid and 8 μM for rifampin; Fig. S1B). The apparent sterilizing activity of rifampin raised the issue of whether other rifamycins would also kill all bacilli present in caseum. Rifapentine, rifabutin, and rifalazil had a casMBC_90_ value of 2 μM (Table 1), lower than that measured for rifampin, as has been shown to be the case in other replicating and nonreplicating assays (23). All three drugs sterilized the caseum at concentrations similar to or lower than those used for rifampin (Fig. 3), despite their significantly lower free fractions in caseum (7.3%, 1.1%, 1.0%, and 0.03% for rifampin, rifapentine, rifabutin, and rifalazil, respectively [24]). Thus, it appears that complete eradication or all persisters is a unique property of the rifamycins and is not affected by high levels of protein binding.

**TABLE 1:** Minimum bactericidal concentrations of first- and second-line TB drugs in caseum, replicating cultures, and two *in vitro* models of nonreplication^*a*^

Antibiotic	Caseum MBC_90_ (μM)	MBC_90_ in replicating culture (μM)	WCC_90_ (μM)	LCC_90_ (μM)
Rifampin	8	0.078	2	2
Isoniazid	>128	0.31–0.63	>128	>128
Pyrazinamide	512	>80	>8,192	>8,192
Moxifloxacin	2	0.31–0.63	10	>128
Linezolid	128	10	>128	>128
Kanamycin	>128	5.0	>128	80
Clofazimine	>128	40	>128	>128
Bedaquiline	32	10	>20	>20
Rifapentine	2	0.078	0.5	10
Rifabutin	2	0.039	0.5	10
Rifalazil^*b*^	2			

a Bactericidal concentrations were measured after 5 days of incubation for bedaquiline, rifapentine, and rifabutin in the WCC_90_ and LCC_90_ determinations (23) and after 7 days in all other assays. Values for MBC_90_ indicate the lowest drug concentration required to kill 90% of the bacterial population. WCC, Wayne cidal concentration; LCC, Loebel cidal concentration.

b Rifalazil is not in clinical use against TB but is included to illustrate the consistency of rifamycin potency in caseum.

We next asked how the casMBC_90_ results would compare to the activity of anti-TB drugs in a standard cidal assay against growing M. tuberculosis in broth and in two cidal assays using nongrowing cultures: the Loebel model of nutrient starved nonreplication (18) and the Wayne model of hypoxia-induced nonreplication (20). Although some of these values have been published elsewhere, most of the nonreplicating cidal assays were repeated and the results were consistent with published data (23) (Table 1 and Fig. S2 and S3). Rifampin and moxifloxacin were among the most potent drugs in replicating cultures, in line with their casMBC_90_ results, but so was isoniazid, which had no activity in the casMBC assay. Rifampin and moxifloxacin were also most active in the Wayne model, although their absolute potency levels were different in the Wayne and casMBC assays (Table 1). Other than kanamycin at 80 μM, the TB drugs had minimal to no activity against oxygen-starved and nutrient-starved nonreplicating bacteria, similarly to what we observed in caseum.

### Lipid accumulation in caseum M. tuberculosis.

**FIG 4:**
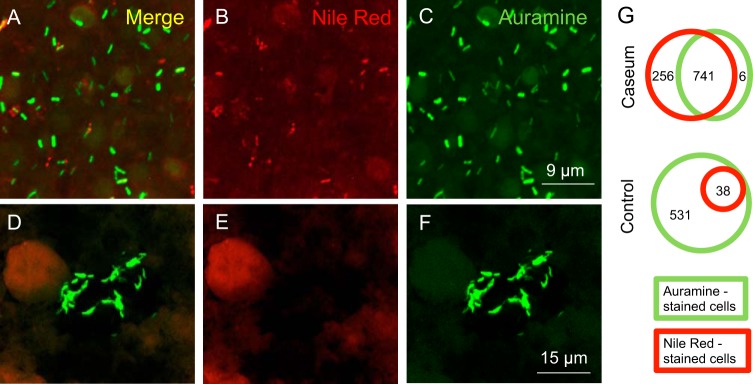
Comparative levels of accumulation of intracellular lipid inclusions and acid-fastness of M. tuberculosis bacilli from caseum and from a replicating culture spiked in synthetic caseum (31). Caseum smears (A to C) and actively replicating M. tuberculosis (D to F) were stained with Auramine O (green; acid-fast stain) and Nile red (red; neutral lipid stain) and examined by confocal laser scanning microscopy (Nikon Eclipse Ti) at the same laser intensity for all samples with Z-stacking to get the depth of the scan field. Scanned samples were analyzed with NIS Elements software for image projection. (A and D) Overlaid image of dual-stained M. tuberculosis. (B and E) Nile red signal only. (C and F) Auramine O signal only. The diffuse red signal in panels E and F is due to staining of lipids present in synthetic caseum. (G) Venn diagrams showing the distribution of auramine-positive and Nile red-positive M. tuberculosis cells in caseum and in an exponentially growing culture spiked in synthetic caseum (control).

Phenotypic antibiotic resistance is associated with the presence of intracellular lipid inclusions (ILIs) irrespective of cell age (25), and ILIs are a signature of M. tuberculosis bacilli found in the sputum of TB patients (26, 27). To determine whether the phenotypic drug resistance of intracaseum bacilli is associated with ILIs, we stained smears of fresh caseum with Auramine O (a diarylmethane dye used as a fluorescent stain for acid-fast bacteria, where it binds to mycolic acids or nucleic acids [28–30]) and Nile red (a lipophilic stain used to localize and quantitate neutral lipid droplets within cells). We compared the level of ILI content of intracaseum M. tuberculosis with that of actively replicating M. tuberculosis smeared in synthetic caseum (31). Confocal laser microscopy revealed a higher frequency of Nile red-stained M. tuberculosis in native caseum smears (Fig. 4A to C) than in replicating culture smears (Fig. 4D to F). In caseum, ILIs were present in both Auramine O-positive and Auramine O-negative M. tuberculosis bacilli (Fig. 4D and E to G), the latter having lost acid-fastness, an indication of cell wall remodeling occurring under the stresses encountered in hostile environment (32). In summary, (i) physiologically distinct (acid-fast-positive and -negative) bacilli are found in caseum, and (ii) ILIs are frequent in both populations.

## DISCUSSION

In this study, we determined for the first time the drug susceptibility of M. tuberculosis bacilli in cavity caseum. Our results demonstrate that intracaseum M. tuberculosis is profoundly tolerant of standard TB drugs. Only rifampin appeared to eradicate all bacilli in caseum, in line with its status as an effective sterilizing agent (33), albeit at a high concentration of 128 μM. The casMBC_90_ of rifampin was 4-fold higher than its MBC_90_ in the Loebel and Wayne nonreplicating assays, indicating that these models of nonreplication are not a perfect surrogate for assessing the potency of TB drugs against caseum bacilli.

Overall, the bacterial burden in *ex vivo* caseum did not change significantly over the 7-day incubation period, indicating that M. tuberculosis bacilli in caseum are largely in a slowly replicating or nonreplicating state. No growth was observed despite disturbance of the *in situ* caseum “environment” brought on by dilution of the matrix and aeration upon excision and homogenization. We excluded the possibility that bacteria were dividing and getting killed with similar kinetics by monitoring chromosome equivalents (CEQ) over time, as determined by quantitative PCR, showing a constant CEQ/CFU ratio over the incubation period (data not shown). Interestingly, the rifampin concentration required to sterilize caseum samples was higher in samples with a higher initial bacillary burden (see Fig. S1 in the supplemental material), indicating that despite consistent MBC_90_ values across caseum batches, the capacity to achieve sterilization at a given concentration is dependent on the size of the starting bacterial burden (Fig. S1). Such an “inoculum effect” has clinical implications and partially explains the long-standing observation that cavitary TB disease—in which high bacillary burdens are found in cavity caseum (34)—is associated with poor prognosis (8, 9).

We have previously measured the concentrations of first- and second-line drugs in the caseum of TB patients at several time points during a 24-h dosing interval (7). Among the 7 drugs tested, only rifampin and moxifloxacin are present in human caseum at or near their casMBC_90_. Following a single rifampin 600-mg dose, peak concentrations of 0.2 to 6.4 μg/g were found in caseum. At the steady state, peak concentrations were not available from this data set, but troughs ranged from 1.4 to 2.9 μg/g at 24 h postdose. Since rifampin was shown to reach higher concentrations in caseum at the steady state than after a single dose (7, 35), it is likely that it approaches or even reaches its casMBC_90_ of 8 μM (6.5 μg/ml) in caseum. Given the high interindividual variability of rifampin pharmacokinetics, additional resected tissues from patients at the steady state are required to confirm this trend.

Moxifloxacin clearly achieves its casMBC_90_ of 2 μM (0.8 μg/ml) in caseum, where we measured steady-state concentrations of 0.6 to 6.6 μg/g in TB patients receiving 400 mg daily (7). This finding could seem surprising given the results of the recent REMox and RIFAQUIN trials where moxifloxacin failed to shorten TB chemotherapy when substituted for either isoniazid or ethambutol in the first-line treatment of drug-sensitive TB (36, 37), indicating a lack of sterilizing activity. One could speculate that drug-mediated killing of intracaseum bacilli is better reflected in early bactericidal activity (EBA) data measuring bacillary burden in sputum than in relapse rates which capture all relevant bacterial populations. Rifampin has modest EBA, but this could be due to its relatively slow partitioning into necrotic foci (7, 35). Despite its low casMBC_90_, moxifloxacin leaves a substantial population of persisters behind (Fig. 2), while rifampin—and all rifamycins—show a steep dose-response curve leading to complete eradication of culturable bacilli (Fig. 2 and 3). In addition, Drusano and colleagues demonstrated an antagonism between rifampin and moxifloxacin against nonreplicating bacilli in the hollow-fiber system (38) which could have contributed to the disappointing results of REMox and even RIFAQUIN if the same antagonism holds true between moxifloxacin and rifamycins in general. In light of these observations, our results suggest that the sterilizing potential of moxifloxacin may be revealed in a regimen that does not include a rifamycin.

Pyrazinamide is regarded as an effective sterilizing agent and an essential member of the short-course regimen (33, 39), but its success as a TB drug remains an enigma because it performs poorly in most *in vitro* potency assays (40, 41). This caseum MBC assay seems to be no exception. Pyrazinamide achieved a 2-log kill level but only at 1 mg/ml (8.2 mM), a concentration not reached within lesions (7). The pH of the rabbit caseum used in this study (7.0 to 7.5, a range similar to results published by others [42–45]), as well as the normoxic to microaerophilic conditions, may have contributed to the modest activity of pyrazinamide. In the necrotic foci of closed granulomas, M. tuberculosis bacilli encounter much lower oxygen tension, leading to reversal of the tricarboxylic acid cycle and secretion of large amounts of succinate (46). This may generate a “halo” of low pH in the direct vicinity of the bacterial cell, creating microenvironmental conditions favorable to the cidality of pyrazinamide and its active metabolite, pyrazinoic acid (47, 48). In a very recent study, pH values of 5 to 5.5 were measured in 8 of 10 cavity caseum samples obtained from resected human lung tissue (49), in contrast with the pH of C3HeB/FeJ mouse (43) and rabbit caseum. It is thus possible that our assay underestimated the activity of pyrazinamide against M. tuberculosis present in the caseum of human lesions, although its preferred target population may be intracellular in the acidified phagolysosome of activated macrophages (43).

Garton et al. observed the presence of ILIs in M. tuberculosis found in sputum samples from TB patients (26, 27). Subsequent studies have established the critical link between the presence of ILIs and the nonreplicating, antibiotic-tolerant phenotype of M. tuberculosis (32, 50), and patients with a greater percentage of lipid body-positive mycobacterial cells in their sputum smear are more likely to have an unfavorable outcome (51). Triacylglycerols (TAG) are a major component of ILIs, and these TAG serve as stored energy (32, 52), supplying the fatty acids which are believed to be the major carbon source of this pathogen in the dormant phase (53, 54). Our observation of increased ILI content in caseum bacilli lends support to this theory and indicates that lipid accumulation is an important feature of M. tuberculosis in this milieu.

As anticipated, a trend toward high drug tolerance was conserved across the three nonreplicating assays, with rifampin being the only agent capable of consistently achieving sterilization. There are, however, qualitative and quantitative differences in the observed drug tolerance data. Overall, intracaseum M. tuberculosis bacilli exhibited higher drug tolerance than nonreplicating M. tuberculosis in the two *in vitro* models employed here (Table 1). Importantly, pyrazinamide was cidal at 512 μM and above in caseum, while it had no detectable activity at up to 8 mM in the Wayne and Loebel assays. Thus, neither the nutrient starvation model nor the oxygen starvation model accurately reproduces the phenotypic resistance of M. tuberculosis in caseum, and *ex vivo* caseum constitutes a unique tool to evaluate drug potency against nonreplicating bacilli in their native caseous environment.

One limitation of this assay resides in the often unpredictable and limited availability of caseum. With the exception of isoniazid and rifampin (Fig. S1), the casMBC data presented here were obtained with frozen/defrosted caseum, after determining that M. tuberculosis bacilli present in freshly explanted and frozen/defrosted caseum exhibited no net growth over the duration of the assay. Transcriptomic studies are in progress with fresh, 4°C-stored, and frozen caseum to investigate the effect of storage on the physiology and metabolism of caseum M. tuberculosis. While the casMBC assay is not amenable to routine testing of large numbers of discovery compounds, it provides a benchmark to validate new surrogate assays which could be developed in synthetic caseum (31). We provide the first experimental evidence that caseum bacilli exhibit profound phenotypic drug resistance, thus joining forces with interindividual PK variability and uneven drug distribution into caseum to create pockets of inadequate drug coverage and potentially leading to relapse or emergence of drug resistance. CasMBC values can be related to the concentrations achieved in the necrotic foci of granulomas and cavities (7), to establish correlations between clinical outcome and lesion-centered PK-PD parameters.

## MATERIALS AND METHODS

### Antimicrobials and reagents.

Moxifloxacin and linezolid were purchased from Sequoia Research Products (Pangbourne, United Kingdom), while rifapentine, rifabutin, pyrazinamide, clofazimine, and isoniazid were purchased from Sigma-Aldrich (MO, USA). Rifampin was purchased from Gold Biotechnology (MO, USA). Kanamycin sulfate was purchased from Fisher Scientific (NH, USA). Stock solutions for all compounds except kanamycin were prepared in dimethyl sulfoxide (DMSO) and stored at 4°C. Kanamycin solution was prepared in water. Rifalazil was synthetized by Bioduro (CA, USA). Bedaquiline was provided by Janssen Research and Development (NJ, USA).

### Rabbit infection model and caseum collection.

All animal studies were performed in biosafety level 3 facilities and approved by the Institutional Animal Care and Use Committee of the New Jersey Medical School, Rutgers University, Newark, NJ, or of the National Institute of Allergy and Infection Disease, NIH, Bethesda, MD. Female New Zealand White (NZW) rabbits (Millbrook Farm, Concord, MA), weighing 2.2 to 2.6 kg, were maintained under specific pathogen-free conditions and fed water and chow *ad libitum*. Rabbits were infected with Mycobacterium tuberculosis HN878 using a nose-only aerosol exposure system (21). The infection was allowed to progress for 12 to 16 weeks prior to necropsy. The right and left lungs were removed and weighed for analytical drug measurement and histopathology. For this study, caseum was collected from rabbits involved in other experiments. Whenever cavities were present, the caseous material was separated from the cellular and fibrotic wall, weighed, and stored in individual 2-ml tubes at −80°C, unless stated otherwise.

### Culture conditions.

Actively replicating cultures of Mycobacterium tuberculosis HN878 were grown in Middlebrook 7H9 media (10% ADC [bovine albumin, dextrose, beef catalase], 0.2% glycerol, 0.05% Tween 80) in vented flasks to an optical density at 600 nm (OD_600_) of 0.3 to 0.6. Cultures were incubated at 37°C on a rotary shaker (250 rpm). In order to generate nutrient-starved nonreplicating (Loebel) cultures of M. tuberculosis, an actively replicating culture was centrifuged at 3,200 rpm and the pellet was washed twice with phosphate-buffered saline supplemented with 0.125% Tween 80 (PBST). The pellet was then resuspended in fresh PBST to the final OD_600_ of 0.2. The bacterial suspension was then incubated at 37°C on a rotary shaker for at least 14 days prior to use in any assays (18). Oxygen-starved nonreplicating (Wayne) cultures were generated in tubes with tight rubber seals which permitted the addition of reagents by needle without disturbing hypoxic conditions. Actively replicating cultures were diluted to the OD_600_ of 0.002 and transferred to these tubes to yield the final headspace air-volume-to-liquid-volume ratio of 3:7. These cultures were stirred constantly at 120 rpm using magnetic stirring bars in a 37°C incubator for 21 days, during which they grew to an approximate OD_600_ of 0.3 to 0.4 before consuming all oxygen and entered the nonreplicating phase. A tube of culture containing methylene blue (1.5 μg/ml) was included in each assay as a visual indicator of oxygen depletion (20).

### MBC assay.

All minimum bactericidal concentration (MBC) assays were carried out in 96-well-plate format. Drug stock solutions (50 mM) were serially diluted in the corresponding vehicle, and 1 μl of each dilution was dispensed in each well. Drugs are not typically tested in MBC assays beyond an upper limit of 80 μM due to solubility issues in aqueous media. However, it was necessary to test much higher concentrations given the apparent drug-tolerant phenotype of M. tuberculosis in caseum. We took advantage of the high macromolecule content of caseum, to which small-molecule drugs bind, particularly the most hydrophobic and least soluble ones (55), thus preventing drug precipitation in the assay medium. Hence, we tested all drugs at the final concentration range of 0.03 to 512 μM, with one exception; PZA was tested at up to 8,192 μM (1 mg/ml) to account for higher clinical exposure.

Frozen caseum was thawed and homogenized in 2 volumes of sterile water by the use of a Fastprep-24 instrument (MP Biomedicals) and 1.4-mm-diameter ceramic (zirconium oxide) beads. Fifty microliters of caseum homogenate was dispensed in each well. The 96-well plates were incubated at 37°C for 7 days. After incubation, caseum homogenate from each well was diluted serially and plated on 7H11 agar in triplicate. Agar plates were incubated for 4 weeks before CFU counts were performed. The caseum MBC_90_ was defined as the concentration of drug required to achieve killing of 90% of M. tuberculosis bacteria contained in the caseum sample. The statistical significance of the differences in CFU counts between day 0 and day 7 (DMSO-only controls) from 10 individual assays was analyzed using paired *t* tests, whereas the overall statistical significance was computed using a multiple *t* test.

To rule out an effect of storage at 4°C and −80°C on the growth kinetics of M. tuberculosis in *ex vivo* caseum, we (i) incubated freshly explanted caseum collected on the day when the experiment was initiated as described above and determined CFU counts per milliliter for up to 28 days at 37°C and (ii) stored fresh (unhomogenized) caseum at 4°C for 15 days prior to initiating growth kinetics experiments at 37°C as described above.

The Loebel 90% cidal concentration (LCC_90_) and the Wayne 90% cidal concentration (WCC_90_) were defined as the drug concentrations required to kill 90% of nutrient-starved and oxygen-starved M. tuberculosis, respectively. LCC_90_ data were determined using a nutrient-starved culture in the 96-well format described above (56). The WCC_90_ was determined by aseptically injecting drugs into the anaerobic culture tubes through the rubber seals. The reaction mixtures were incubated for 7 days for both assays.

### pH measurement.

Caseum was homogenized in sterile water (3-fold dilution), and the pH of the homogenate was measured using pH indicator test strips from two different suppliers: Micro Essential Laboratory (NY, USA) and Whatman (Maidstone, United Kingdom).

### Auramine-Nile red labeling.

Three-fold-diluted rabbit caseum homogenate and actively replicating HN878 M. tuberculosis grown in 7H9 medium and spiked in synthetic caseum prepared as described previously (31, 55) were spread evenly on the surface of glass slides using 10-μl inoculating loops, air dried, and fixed with paraformaldehyde. The slides were submerged in 10% formalin for 1 h to sterilize them. The smears were stained with Auramine and Nile red as previously described (26). Briefly, the smears were stained with Auramine O (Remel; KS, USA) for 15 min, decolorized with 3% acid alcohol (Remel) for 15 min, and then labeled with Nile red (Sigma) (10 μg/ml in ethanol) for 10 min. The smears were then stained with KMnO_4_ for 1 min, washed, and mounted with ProLong Gold Antifade reagent (Invitrogen, CA, USA). Images were acquired by confocal laser microscopy at 561 nm (excitation) and 582 to 622 nm (emission) for the red channel and 489 nm (excitation) and 522 to 542 nm (emission) for the green channel, using an A1 confocal microscope (Nikon, Tokyo, Japan), and analyzed using NIS Elements software (Nikon). A spectrum detection system was used to isolate specific wavelengths and avoid overlap in the fluorescence emission data. For quantitative comparison of data from the Nile red-positive and auramine-positive cells in native caseum and replicating cultures, the fluorescence of >500 individual cells was measured. The fluorescence intensity of the background of the image was subtracted from the pixel value measured for each fluorescent cell. Three-dimensional reconstruction and deconvolution of areas of interest were performed on 12 to 25 optical sections obtained at 0.250-μm or 0.150-μm intervals (57).

## Supplementary Material

Supplemental material
