# Supplementary material for: Extreme Drug Tolerance of Mycobacterium tuberculosis in Caseum
Source: Antimicrob Agents Chemother. 2018 Jan 25;62(2):e02266-17. doi: 10.1128/AAC.02266-17 (PMC5786764; doi:10.1128/AAC.02266-17)
Supplement: Supplemental material [file supp_62_2_e02266-17__index.html]

Supplemental material 

# Extreme Drug Tolerance of Mycobacterium tuberculosis in Caseum

## Supplemental material

- Supplemental file 1 -

  Supplemental Figures S1 to S3

  PDF, 135K
