# Supplementary material for: Extreme Drug Tolerance of Mycobacterium tuberculosis in Caseum
Source: Antimicrob Agents Chemother. 2018 Jan 25;62(2):e02266-17. doi: 10.1128/AAC.02266-17 (PMC5786764; doi:10.1128/AAC.02266-17)

Supplemental Figures

Figure S1. Net growth and drug susceptibility of Mtb in fresh caseum and caseum stored at 4°C. (A) To rule out an effect of storage at 4°C and -80°C on the growth kinetics of Mtb in ex vivo caseum (potentially due to a metabolic shift upon storage at 4 or -80°C), we compared the CFU kinetics of fresh caseum (harvested from rabbits on the day when the experiment was initiated) and caseum stored non-homogenized at 4°C for 15 days. Separate batches of caseum were obtained from three different rabbits and either used fresh on the day of necropsy or stored at 4°C, prior to initiating growth kinetic experiments. Mtb bacilli in fresh and 4°C-stored caseum remained viable and showed no net-growth over the incubation periods at 37°C, similar to caseum stored at -80°C prior to initiating MBC experiments (Fig. 1). To rule out the possibility that the no-net-growth observed by CFU could be the result of balanced growth and death of Mtb bacilli in ex vivo caseum, we also measured chromosome equivalents (CEQ) over an incubation period of 7 to 14 days at 37°C, using quantitative PCR. The CEQ values remained constant over time, both in fresh caseum and caseum stored at 4°C for 15 days prior to initiating incubation at 37°C (data not shown); (B) Bactericidal activity of rifampicin and isoniazid in caseum collected 3 days prior to the assay and stored at 4°C. Data in (A) and (B) are expressed as average CFU per ml of homogenized caseum (n=3) plotted on a log scale.

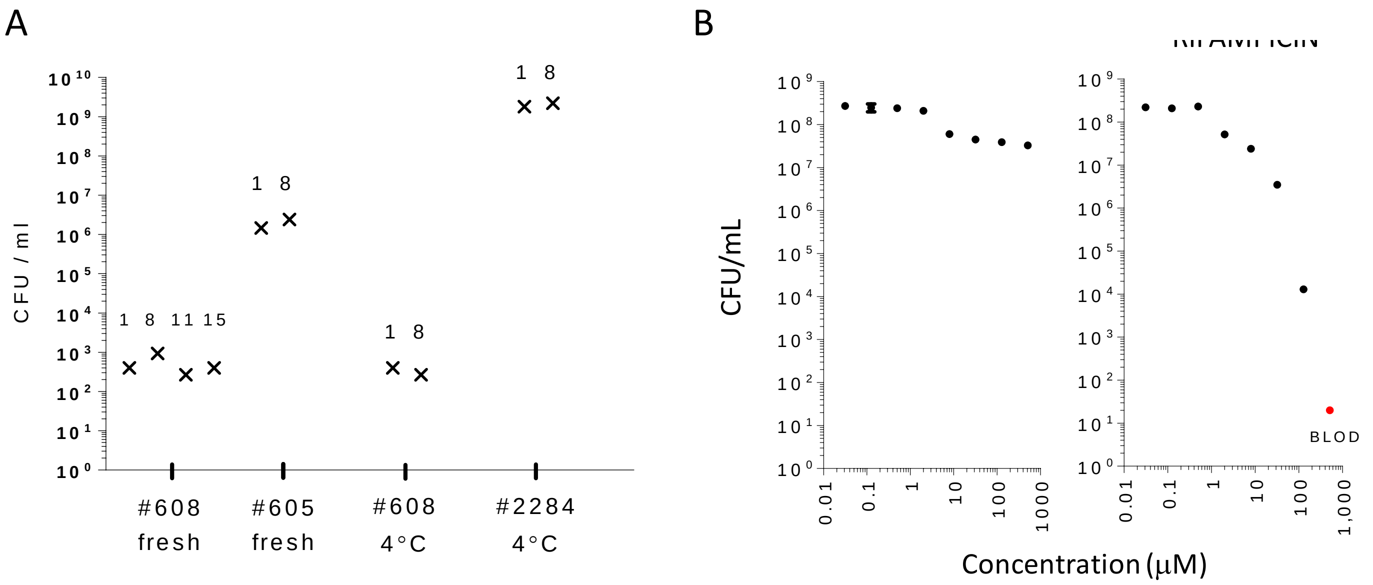

Figure S2. Bactericidal activity of seven standard TB drugs in nutrient-starved non-replicating *M. tuberculosis* cultures. Data are expressed as CFU per ml and are plotted on a log scale. Error bars indicate standard deviations and are sometimes hidden.

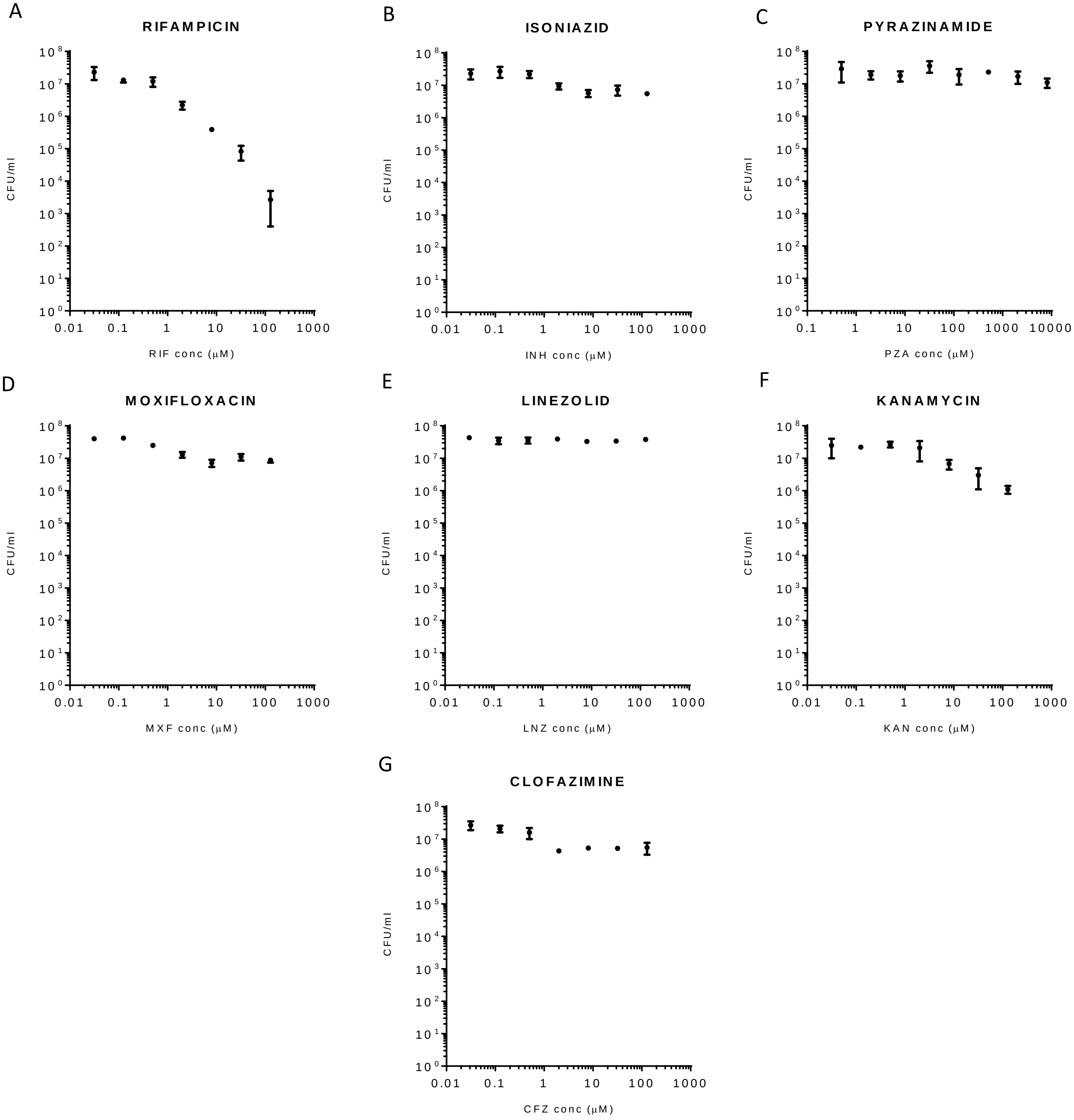

Figure S3. Bactericidal activity of seven standard TB drugs in oxygen-starved non-replicating *M. tuberculosis* cultures. Data are expressed as CFU per ml and are plotted on a log scale. Error bars indicate standard deviations and are sometimes hidden.

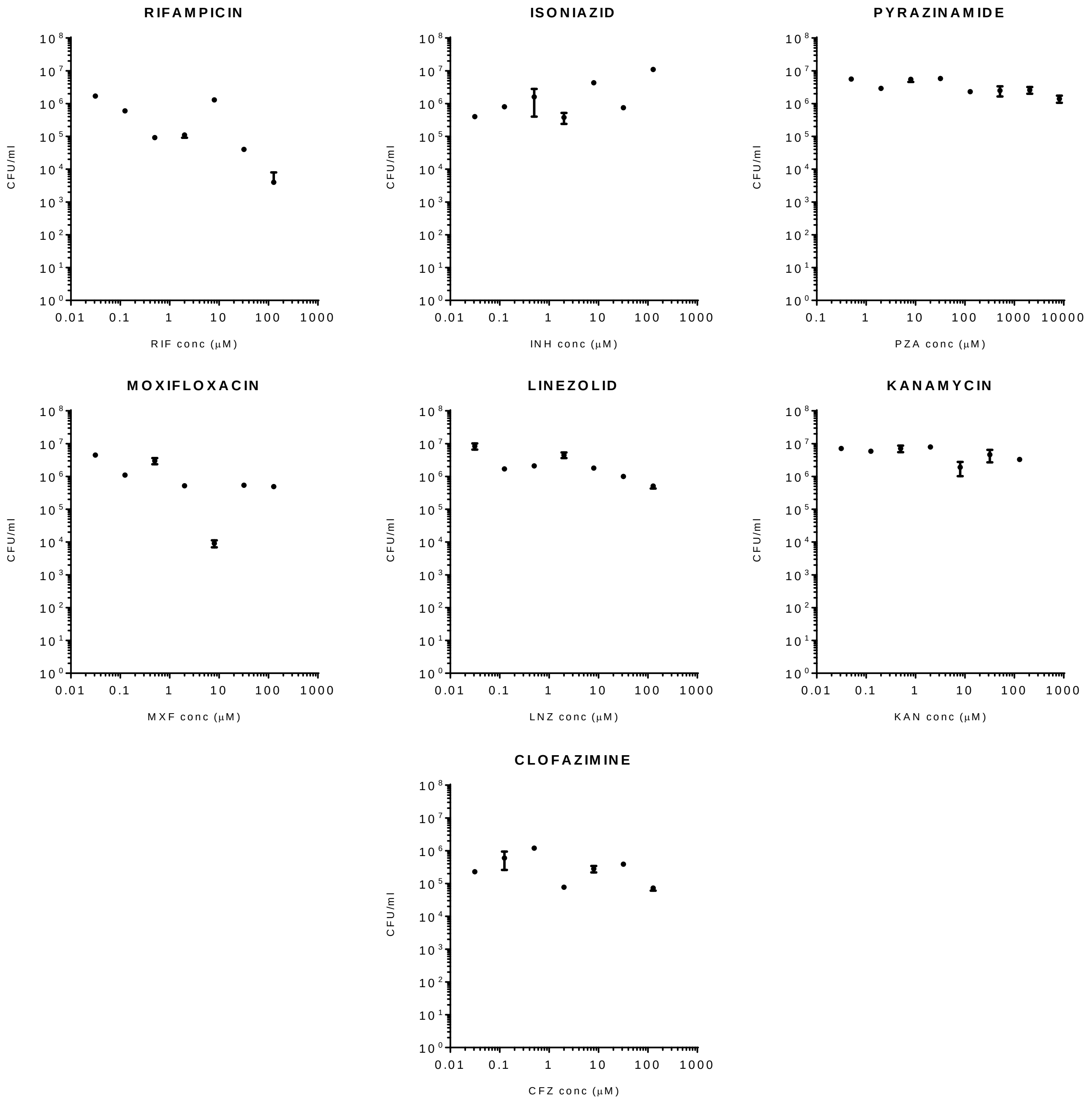

Supplement: Supplemental material [file AAC.02266-17_zac002186897s1.pdf]
